# Adherence to treatment guidelines for acute diarrhoea in children up to 12 years in Ujjain, India - a cross-sectional prescription analysis

**DOI:** 10.1186/1471-2334-11-32

**Published:** 2011-01-28

**Authors:** Deepali Pathak, Ashish Pathak, Gaetano Marrone, Vishal Diwan, Cecilia Stålsby Lundborg

**Affiliations:** 1 Division of Global Health (IHCAR), Department of Public Health Sciences, Karolinska Institutet, Stockholm, Sweden; 2 Consultant Paediatrician, Grasim Trust's G.D. Birla Memorial Hospital, Ujjain, India; 3 Department of Paediatrics, R.D. Gardi Medical College, Ujjain, India; 4 Department of Public Health and Environment, R.D. Gardi Medical College, Ujjain, India

## Abstract

**Background:**

Diarrhoea accounts for 20% of all paediatric deaths in India. Despite WHO recommendations and IAP (Indian Academy of Paediatrics) and Government of India treatment guidelines, few children suffering from acute diarrhoea in India receive low osmolarity oral rehydration solution (ORS) and zinc from health care providers. The aim of this study was to analyse practitioners' prescriptions for acute diarrhoea for adherence to treatment guidelines and further to determine the factors affecting prescribing for diarrhoea in Ujjain, India.

**Methods:**

This cross-sectional study was conducted in pharmacies and major hospitals of Ujjain, India. We included prescriptions from all practitioners, including those from modern medicine, Ayurveda, Homeopathy as well as informal health-care providers (IHPs). The data collection instrument was designed to include all the possible medications that are given for an episode of acute diarrhoea to children up to 12 years of age. Pharmacy assistants and resident medical officers transferred the information regarding the current diarrhoeal episode and the treatment given from the prescriptions and inpatient case sheets, respectively, to the data collection instrument.

**Results:**

Information was collected from 843 diarrhoea prescriptions. We found only 6 prescriptions having the recommended treatment that is ORS along with Zinc, with no additional probiotics, antibiotics, racecadotril or antiemetics (except Domperidone for vomiting). ORS alone was prescribed in 58% of the prescriptions; while ORS with zinc was prescribed in 22% of prescriptions, however these also contained other drugs not included in the guidelines. Antibiotics were prescribed in 71% of prescriptions. Broad-spectrum antibiotics were prescribed and often in illogical fixed-dose combinations. One such illogical combination, ofloxacin with ornidazole, was the most frequent oral antibiotic prescribed (22% of antibiotics prescribed). Practitioners from alternate system of medicine and IHPs are significantly less likely (OR 0.13, 95% CI 0.04-0.46, P = 0.003) to prescribe ORS and zinc than pediatricians. Practitioners from 'free' hospitals are more likely to prescribe ORS and zinc (OR 4.94, 95% CI 2.45-9.96, P < 0.001) and less likely to prescribe antibiotics (OR 0.01, 95% CI 0.01-0-04, P < 0.001) compared to practitioners from 'charitable' hospitals. Accompanying symptoms like the presence of fever, pain, blood in the stool and vomiting significantly increased antibiotic prescribing.

**Conclusion:**

This study demonstrated low adherence to standard treatment guidelines for management of acute diarrhoea in children under 12 years in Ujjain, India. Key public health concerns were the low use of zinc and the high use of antibiotics, found in prescriptions from both specialist paediatricians as well as practitioners from alternate systems of medicine and informal health-care providers. To improve case management of acute diarrhoea, continuing professional development programme targeting the practitioners of all systems of medicine is necessary.

## Background

Acute gastroenteritis continues to be a leading cause of mortality and morbidity in the paediatric population globally, and is responsible for death of 2.5 million under-five children every year [1]. Viral pathogens such as *rotavirus *account for 70-80% of all diarrhoeal episodes [2]. In India, diarrhoeal diseases are the second leading cause of child mortality (20%) after acute respiratory infections (30%) [3].

The joint statement by WHO and UNICEF in 2004 recommended the use of low osmolarity oral rehydration solution (ORS) along with zinc for 14 days as an adjunct therapy to decrease diarrhoeal deaths among the world's most vulnerable children [1,4]. Low osmolarity ORS contains 75 mEq/L of sodium, 75 mmols/L of glucose and has an osmolarity of 245 mOsmols/L. New low osmolarity ORS is the single universal ORS solution for all types of diarrhoea and for all ages. Zinc has been found to reduce the incidence, frequency, severity and persistence of diarrheal episodes in children older than six months [5].

Based on a meta-analysis of randomised placebo controlled trials, the Indian Academy of Paediatrics (IAP) published guidelines for management of acute diarrhoea in 2004 [6]. The guidelines focussed on the use of low osmolarity ORS, zinc in acute diarrhoea; antibiotic use in dysentery; and management of diarrhoea in severely malnourished children. These guidelines were further revised in 2006 [7]. The key points of the guidelines are presented in Table 1.

**Table 1:** The Indian Academy Of Paediatrics Recommendations for the treatment of acute diarrhoea in children [6,7]

	Drug	Recommendations
1.	Low osmolarity ORS	Universal ORS for all ages in all types of diarrhoea.
2.	Zinc supplementation	A uniform dose of 20 mg of elemental zinc should be given during the period of diarrheal and for 7 days after cessation of diarrheal to children older than 3 months.
3.	Pre-biotics, probiotics and Racecadotril	Presently insufficient evidence to recommend in the treatment of acute diarrhoea.
4.	Antiemetics^a^	Reserved for children in whom the vomiting is severe, recurrent and interferes with ORS intake.
5.	Antibiotics^b^	To be used only for acute bloody diarrhoea (stools with visible blood); recommended in 2004 guidelines only [6]. The dug of choice is Co-trimoxazole if local prevalence of resistance in Shigella is less than 30%; nalidixic acid if resistance exceeds 30%, norfloxacin, ciprofloxacin or a third generation cephalosporin must be used as second and third line drugs.

^a^A single dose of domperidone is recommended in children with severe vomiting.

^b^Antibiotics are not indicated for children with acute diarrhoea and no visible blood in stools, with pus cells on stool microscopy. IAP does not recommend routine stool examination in children with acute diarrhoea. *Entamoeba histolytica *and helminths are very rare causes of acute diarrhoea in children thus; metronidazole and antihelminthics have no role. Aminoglycosides like gentamicin and amikacin are ineffective in the management of acute bloody diarrhoea.

The Government of India accepted the revised guidelines for nationwide use in 2007 [6,7]. Dissemination of the guidelines is carried out both at a national and at a local level through mass media campaigns arranged by the government. The IAP has been organizing sensitizing workshops for paediatricians since 2004 for the states with the highest disease burden of diarrhoea (Bihar, Madhya Pradesh, Uttar Pradesh and Rajasthan).

It has been found that adherence to treatment guidelines for the management of common childhood illnesses such as diarrhoea and respiratory tract infections is low worldwide [8,9] and in India [10]. Drugs such as antibiotics, probiotics and racecadotril (an anti-secretory drug that reduces the secretion of water and electrolytes in the intestine) are increasingly being prescribed for acute diarrhoea in India. Alternate systems of medicine such as Ayurveda, Yoga and Naturopathy, Unani, Siddha, Homeopathy (AYUSH) and informal health-care providers (IHPs) coexist to provide health care. Although they are not legally permitted to do so, many of these providers prescribe or dispense drugs of the allopathic system of medicine [11]. Their services are frequently used in rural areas, due to cultural and socioeconomic reasons. There have been no published studies to determine the extent to which such providers follow treatment guidelines in India [12].

This study was conducted in the city of Ujjain, Madhya Pradesh, India. The main aim was to determine the levels of adherence to treatment guidelines for acute diarrhoea in children up to 12 years seen in drug prescriptions, and further to explore the factors affecting prescribing of ORS with zinc and antibiotics.

## Methods

### Study area and setting

A cross-sectional quantitative study including major pharmacies and hospitals was conducted in Ujjain city; headquarter of Ujjain district, Madhya Pradesh, India. The Ujjain district has a population of 1,710,000 and an infant mortality rate of 52 per 1000 live births.

Ujjain city has more than 300 modern medicine graduates and post-graduates in different fields of medicine. They provide health care in government run hospitals or dispensaries, charitable hospitals or through private clinics. Many of the practitioners in government and charitable hospitals are allowed to work in private clinics outside of their official hours of work. Practitioners from other formal systems of medicine such as Ayurveda and Homeopathy (110 in number) and informal health-care providers (800 in number) outnumber the allopathic practitioners. Most (82%) of the informal health-care providers are based in the rural areas [13].

There are about 300 pharmacies in the city, consisting of retailers (75%) and stock dealers (25%). A typical pharmacy has one main pharmacist and three to four pharmacy assistants or trainees working on shifts.

### Sample size

For sample size calculation we considered ORS prescribing as the primary outcome and prescribing of "ORS and zinc" and "antibiotics" as the secondary outcomes. We chose ORS prescribing as the primary outcome as there were no previous estimates of zinc prescribing for the region. Sample size calculation was done based on a pilot study, which showed a prescription rate of 50% for ORS. Thus, assuming 50% as the basic percentage of prescribing of ORS and requesting a 95% confidence interval for the proportion with width no higher than 15%, the minimum sample size needed is 170. A conservative estimate of design effect of 4 was considered appropriate [14]; this gave a minimum sample size needed of (170*4) 680 patients.

### Data collection instrument

The data collection instrument (Table 2) contained demographic information of the patient, practitioners' information, and a brief description of the diarrheal episode and prescription given for that particular episode of diarrhoea. For all drugs except antibiotics a list of commonly used commercial names was included in the form. For antibiotics a list of generic names was used. The practitioners were categorised according to their qualification and duration of practice (years in practice after graduation or post-graduation). The hospitals were categorised as free (attached to medical college and with no fee for consultation or medicines), charitable (nominal fee for consultation with or without free medicines) and private (high fee for consultation).

**Table 2:** Data collection instrument

**Patient information**	**Name**
	Age
	Sex
	Address
	Outpatient/inpatient
	Date of prescription Pharmacy code Hospital code
Practitioner's information	Qualification
	Duration in practice
Information on Diarrhoeal episode	Duration of diarrhoea
	Presence of blood
	Presence of fever
	Presence of vomiting
	Presence of pain in stomach
Prescription given	ORS
	Zinc
	Antibiotics
	Probiotics
	Racecadotril
	Miscellaneous drugs (including antiemetic)

### Data collection

Ujjain city was divided into four zones based on the commonly used geographic divisions. In each zone, the top five pharmacies based on retail sales (according to the Ujjain Druggists and Chemists Association) and the major public and private hospitals (defined as a hospital with more than 100 beds) in the city were approached to participate in the study.

**Figure 1:**
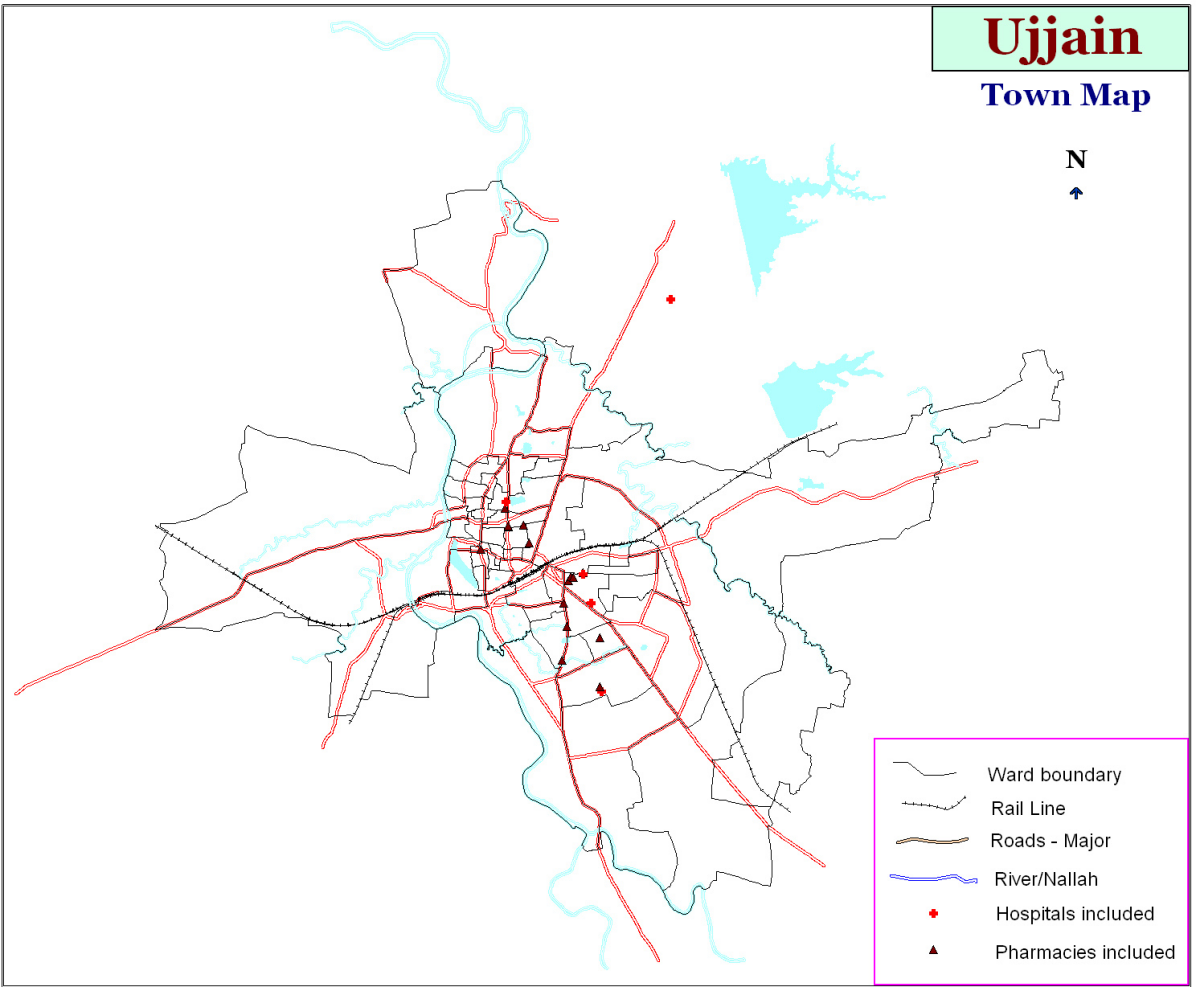
GIS map of Ujjain city showing the participating pharmacies and hospitals

Seventeen of the top twenty pharmacies and five of six major hospitals consented for the study. The participating hospitals and pharmacies are shown in Figure 1. Two pharmacy assistants at each pharmacy were identified to assist in the collection of information from the outpatient prescriptions. They received training on filling the data collection form, as well as to ask questions about the current diarrheal episode (duration of diarrhoea, fever, vomiting, presence of blood in stools). Where possible all data was collected from prescriptions, but the participating pharmacy assistants were trained to ask questions pertaining to the diarrhoeal episode if such information was incomplete. In hospitals, resident medical officers were also trained for transferring the information from in-patient case sheets of patients with acute diarrhoea to the data collection instrument. A pilot study was done in one hospital and two pharmacies for two weeks. No change in the data collection instrument was found to be necessary after the pilot. The prescriptions from the pilot study were not included in the final sample. Data collection for the main study was done from 1^st ^June to14^th ^August 2009.

Only complete prescriptions containing acute diarrhoea case history and treatment details were included in the study. Prescriptions from same patient were not entered twice. Data collection was validated through weekly visits to the participating pharmacies and hospitals by the first author.

### Data management and analysis

The data was entered in Epidata Entry (version 3.1); Stata 10.0 (Stata Corp. College Station, Texas, USA) was used for data analysis. Descriptive statistics for all the principal variables were calculated. Frequencies and percentages were used for categorical variables. Two multivariate stepwise logistic regression models: one for ORS with zinc and other for antibiotics (prescribed versus not prescribed) as outcome measures were computed. We controlled the outcome variables for design effect due to intra cluster correlation (ICC) within the pharmacies and hospitals. ICC is a measure of the correlation between outcome values ("ORS with Zinc" or "antibiotics") in prescriptions from the same cluster (hospitals or pharmacies). If all the prescriptions from the same cluster have identical outcome values, ICC is equal to 1. ICC is used in calculating the design effect, a measure of how the sample size is affected by the clustering of prescriptions, compared to a random sample. It was essential to adjust for design effect in our analysis because the prescriptions originating from the same pharmacies or hospitals (clusters) were likely to be similar. As a result, in a clustered sample such as ours, selecting an additional prescription from the same cluster adds less new information than a random sample. Since the sample is not as varied as it would be in a random sample, the effective sample size is reduced. The loss of effectiveness by the use of cluster sampling, instead of simple random sampling, is the design effect. The design effect shows the effect of the study design on the estimate's variance and increases with within-cluster homogeneity. In our study the prescriptions were collected from 17 pharmacies and 5 hospitals, so we specified the survey design in Stata command in order to adjust the variances estimates, confidence intervals (CI) and P-values. The design effect was calculated for both pharmacies and hospitals using the formula DE = 1 + ICC (m-1), (where m is the average number of observations in each cluster).

The independent variables were practitioners' qualification (post graduates/allopathic graduates/Others - alternate systems of medicine and informal health-care providers), duration of practice (0 to10 years/more than 10 years), age of the child (0 to < 1yr/1 to < 5yrs/5 to ≤ 12yrs), gender (male/female), locality (urban/rural), duration of diarrhoea (up to 7 days/>7 days), fever (yes/no), stomach pain (yes/no), vomiting (yes/no), practice setting (free/charitable/private), patient setting (in-patient/out-patient) and month of prescription (June/July/August). Covariates found significant in bivariate analysis (Chi-square test) at a level of P < 0.20 were included in the model and removed using a backwards stepwise method (Wald test with removal level of significance of P < 0.10). Odds ratios (OR) and their 95% confidence intervals (CI) were also computed. We applied Bonferroni correction for OR's because of the double comparison of independent variables with two outcomes. A value of P < 0.05 was considered statistically significant in the final models. The test of significance was two-sided.

### Ethics

The ethics committee of Ujjain Charitable Trust Hospital and Research Centre and R.D. Gardi Medical College, Ujjain approved the study (approval number 63). Informed consent was attained from the participating pharmacies and hospitals, and from the parents of children whose prescriptions were included in the study. The names of practitioners were not included in the data collection instrument for ethical reasons.

## Results

A total of 843 completed prescriptions were collected. Table 3 shows the distribution of patients' and practitioners' characteristics. Most prescriptions were for children under five years of age (64%) with a male predominance (57%). Most of them came from the rural areas of Ujjain (66%) and presented in the outpatient department (73%) in the early rainy season (July 2009). Almost all (97%) of the patients had acute diarrhoea for up to 7 days duration, with fever reported in 52% and bloody stools in 11%. Most (68%) prescriptions were from paediatricians. Most (68%) prescriptions originated from doctors with less than 10 years of practice (either after graduation or post-graduation). Rural patients accounted for 76% of all the hospital admissions. Of the total prescriptions for intravenous fluids (IVF's), 71% were for rural patients. A higher proportion (66%) of the rural patients were prescribed antibiotics for acute diarrhoea.

**Table 3:** Distribution of patients' and practitioners' characteristics in the prescriptions of acute diarrhoea collected by 17 pharmacies and 5 major hospitals of Ujjain, India (n = 843)

Patient characteristics		Number	Percentage
Age	0 to < 1 year	221	26
	1 to < 5 years	323	38
	5 to ≤12 years	299	36
Sex	Male	481	57
	Female	362	43
Locality	Urban	289	34
	Rural	554	66
Patient setting	Outpatient	612	73
	In patient	231	27
Month of prescription	June	262	31
	July	516	61
	August	65	8
**Diarrhoea Information**			
Duration of diarrhoea	Up to 7 days	819	97
	More than 7 days	24	3
Associated symptoms	Presence of fever	442	52
	Presence of pain in stomach	183	22
	Presence of vomiting	348	41
	Presence of blood in stools	89	11
**Practitioner's characteristics**			
Prescriptions according to practitioner's qualifications	Paediatricians (Post-graduate degree or diploma)	571	68
	Allopathic graduates (MBBS)	101	12
	Others (alternate system of medicine and informal health-care providers)	171	20
Duration in practice	0 - 10 years	569	68
	More than 10 years	274	32
Practice setting	Free	170	20
	Charitable	141	17
	Private	532	63

### Pattern of prescription for acute diarrhoea

The proportion of ORS prescriptions in the study was 58% (Table 4). ORS with zinc was prescribed in 22% of cases but these prescriptions contained other drugs (antibiotics, probiotics, racecadotril and antiemetics with the exception of Domperidone). Out of the 843 prescriptions, 71% included one or more antibiotics. Broad-spectrum antibiotics were used and often in illogical combinations. One such combination, ofloxacin with ornidazole, was the most frequent oral antibiotic prescribed (22% of the prescriptions containing antibiotics). Drugs for fever, stomach pain and vomiting were found in 69% of the prescriptions. Probiotics were found in 68% and racecadotril in 19% prescriptions. All patients that had been admitted to one of the hospitals, and 2% of outpatients, were prescribed intravenous fluids (IVFs). Ondansetron was prescribed more frequently in the free hospitals.

**Table 4:** Distribution of ORS, zinc, antibiotics and other drugs in the prescriptions for acute diarrhoea in Ujjain, India (n = 843)

	Number	Percentage	**Percentage range between clusters**^a^
ORS	487	58	19-99%
ORS with zinc	188	22	0-53%
ORS with zinc and antibiotics	88	10	0-41%
Zinc only	228	27	0-53%
Antibiotics	602	71	8-100%
Probiotics	574	68	
Racecadotril	160	19	
Miscellaneous (for fever, pain in stomach, vomiting)	589	69	
IVFs	241	29	
IVFs with ORS	120	14	
IVFs without ORS	121	14	

^a^Percentages were calculated separately for each cluster (pharmacy/hospital); "Percentage between clusters" reports the smallest and largest of these percentages.

### Factors affecting co-prescribing of ORS along with zinc

The multivariate stepwise logistic regression model showed that there was a significant association between prescribing ORS with zinc and the practitioner's qualification. The practitioners from alternate systems of medicine and the IHPs prescribed ORS with zinc significantly less often than the modern medicine graduates and postgraduates practitioners (Table 5). Practitioners working in the free hospitals prescribed ORS with zinc significantly more often than those working in the charitable hospitals. However, no significant difference was found between those working in the private and charitable hospitals. The odds of prescribing ORS with zinc were significantly greater in the presence of stomach pain (OR 3.54, 95% CI 1.98-6.32; P < 0.001). Duration of practice, age of the child, presence of fever, blood in stools and occurrence of vomiting was not shown to significantly affect co-prescribing of ORS with zinc.

**Table 5:** Distribution of the factors affecting prescription of ORS and zinc in children up to 12 years with acute diarrhoea in Ujjain, India

Factors affecting ORS with zinc prescription	Categories	ORS with zinc (n = 188)				
		n	%^#^	Adjusted OR	95% CI	P Value
Practitioner's qualification	Postgraduates	571	26	1	-	-
	Allopathic graduates	101	37	0.89	0.62-1.27	0.494
	Alternate medicine and informal health-care providers	171	2	0.13*	0.04-0.46	0.003
Duration in practice	0 to 10 years	569	28	1	-	-
	More than 10 years	274	11	0.61	0.35-1.07	0.081
Practice setting	Charitable	141	25	1	-	-
	Free	170	53	4.94*	2.45-9.96	< 0.001
	Private	532	12	0.56	0.23-1.38	0.196
Presence of associated symptom	Pain in stomach (no)	660	20	1	-	-
	yes	183	31	3.54*	1.98-6.32	< 0.001

* P < 0.05

Results adjusted for age of the child and sex.

n=number in each category and %^# ^= proportion of those providers who prescribe ORS and zinc

### Factors affecting antibiotic prescribing

Patients with prescriptions from practitioners of alternate systems of medicine and informal health-care providers received antibiotics significantly more often than patients with prescriptions from postgraduate practitioners. No significant difference was found between prescriptions from allopathic graduates and postgraduate practitioners (Table 6). The odds of prescribing antibiotics were significantly higher in the presence of fever (OR 3.32, 95% CI 1.82-6.07; P < 0.001), stomach pain (OR 7.25, 95% CI 1.81-28.94; P = 0.007) and blood in stools (OR 9.93, 95% CI 3.65-27.01; P < 0.001). Duration of practice, practice setting, patient setting, age of the child and duration of diarrhoea did not significantly affect antibiotic prescribing.

**Table 6:** Distribution of the factors affecting prescription of antibiotics in children up to 12 years with acute diarrhoea in Ujjain, India

Factors affecting antibiotic prescription	Categories	Antibiotics (n = 602)				
		n	%^#^	Adjusted OR	95%CI	P Value
Practitioner 's qualification	Postgraduates	571	70	1	-	-
	Allopathic graduates	101	39	1.32	0.52-3.35	0.548
	Alternate medicine and informal health-care providers	171	95	3.21*	1.19-8.65	0.023
Practice setting	Charitable	141	74	1	-	-
	Free	170	8	0.01	0.01-0.04	< 0.001
	Private	532	91	2.54	0.91-7.09	0.073
	Fever (no)	401	64	1	-	-
Associated symptoms	yes	442	78	3.32*	1.82-6.07	< 0.001
	Pain in stomach (no)	660	66	1	-	-
	yes	183	92	7.25*	1.81-28.94	0.007
	Blood in stools (no)	754	0.4	1	-	-
	yes	89	97	9.93*	3.65-27.01	< 0.001

* P < 0.05

Results adjusted for age of the child and sex.

n=number in each category and %^# ^= proportion of those providers who prescribe antibiotics.

### Intra-cluster correlation (ICC) and design effect

ICC for the outcome variables was estimated and was 0.27 for ORS and Zinc prescribing, and 0.56 for antibiotics prescribing.

The design effect, obtained with the formula DE = 1+ICC (m-1), (where m is the average number of observations in each cluster, 37 in our study) is 11 and 21 respectively for "ORS and zinc" and antibiotics prescribing.

### Compliance with guidelines

Only six prescriptions for acute diarrhoea were found to include both ORS and Zinc, without probiotics, antibiotics, racecadotril or antiemetics (except Domperidone which could be prescribed for vomiting).

## Discussion

In this study, the management of acute watery diarrhoea more frequently included the use of ORS (58%) as compared with a previous Indian study, where ORS was prescribed to only 22% of the children [10]. The correct treatment according to the guidelines is ORS with Zinc, without the use of probiotics, antibiotics, racecadotril or antiemetics (with the exception of Domperidone for severe vomiting). This was prescribed in only six out of 843 prescriptions. ORS with zinc was prescribed in 22% of cases, but these prescriptions also contained other drugs (antibiotics, probiotics, antiemetics and racecadotril), which are not recommended in the guidelines. The improvement in the proportion of cases in which ORS was prescribed may be due to the increasing awareness amongst the practitioners and the community about its importance in treating diarrhoea. This increased awareness may be due to mass media campaigns about the importance of ORS. These campaigns have not focussed on the benefits of zinc, so there may be less awareness of these among practitioners as well as the community. This issue warrants further exploration by both qualitative and quantitative studies.

Randomised controlled trials from low and middle income countries have shown that zinc is a cost effective intervention, along with ORS, in reducing morbidity from diarrhoea [7], but knowledge of health care providers about its proper use is lacking. A retrospective study [15] carried out in a private tertiary care hospital of Chennai, India, to study the pattern of prescribing of zinc and antibiotics for acute watery diarrhoea showed that the use of zinc had increased to 75% over a three-year period. This was accompanied by a decline in the use of antibiotics to below 30%, which was achieved through education of health care workers on the use of zinc. This suggests the need for education of the benefits of using zinc in acute diarrhoea to decrease the duration and severity of diarrhoea, as well as appropriate antibiotic use. In the present study antibiotics were prescribed for 71% of patients, compared with the WHO recommendations of around 20%.

There was deviation from the guidelines with regard to probiotic and racecadrotil use in our study. Probiotics were prescribed in 68% of cases, and racecadotril in 19% of cases; according to the guidelines neither of these should have been prescribed at all.

Some possible reasons for the high rate of prescribing of drugs not recommended in the guidelines are: *first, *the natural history of diarrhoeal episode in which treatment with ORS neither shortens the duration of diarrhoea nor decreases the stool volume. There is a problem with the 'image' of ORS and zinc as effective treatment options. Up to 40% of children revisit the doctor as they have significant stool frequency for more than 4 days, despite taking ORS [16]. As ORS does not reduce diarrhoea duration, private paediatricians and other private health care workers look for alternatives to reduce the duration of diarrhoeal episodes [17,18]. *Second*, vomiting is often an important issue for parents as well as physicians during ORS therapy (ORT). The cause of vomiting in acute diarrhoea is usually hypokalaemia. Vomiting can be both caused and aggravated by incorrectly prepared ORS (hyperosmolar ORS). Parents may be discouraged to continue ORT because of this vomiting, leading to a failure of oral rehydration [19]. Severe vomiting should lead to prescription of domperidone and not other drugs according to the IAP guidelines. However, the perceived ineffectiveness of ORT may have lead to a growing interest in adjunctive treatments such as antibiotics, probiotics and racecadotril [20]. These drugs are also highly marketed by pharmaceutical companies, with incentives for practitioners, playing a major role in prescribing. *Third*, the presence of fever increases antibiotic prescribing even though most diarrhoeal episodes are of viral origin [21]. Consequently fever remains an important and unjustified reason for prescribing antibiotics for many self-limiting infections. *Fourth*, rural patients contributed to hospital admissions for acute diarrhoea more frequently in our study. They received 61% of the total antibiotic prescriptions and 71% of the total intravenous rehydration prescribed. This may reflect: a) referral of sicker patients to the city; b) demand for parenteral therapy for acute diarrhoea by patients; and c) the practitioners' preference to admit rural patients in order to monitor them. Starting IVFs and antibiotics for these patients can 'justify' the admission [22]. *Fifth*, most prescriptions that contained ORS with zinc were from practitioners working in the free hospital attached to medical college. Interestingly it was also the hospital with the least antibiotic prescribing (7.6%). However, there was deviation from the guidelines in terms of antiemetic (ondensetron) prescribing. Most of the prescriptions in the free hospital attached to the medical college came from fresh medical graduates or post-graduate practitioners working in the paediatric department. Working in an academic institution with access to updated information might be responsible for the low antibiotic prescribing seen [23]. Practitioners working in the free and the charitable hospitals were more likely to prescribe in accordance with the guidelines than those in the private sector. A reason might be that the private physicians needed to provide symptomatic relief faster for the fear of losing patients to another physician, and therefore used more drugs for fever, stomach pain, vomiting and antibiotics [24]. This is illustrated in the present study by the high ICC for antibiotic prescribing, which indicates the homogeneity within each cluster.

The AYUSH practitioners and the IHPs prescribed ORS with zinc less frequently and were found to prescribe antibiotics more frequently, underlining that these practitioners need to be made aware of standard treatment guidelines if they have to practice modern medicine, and must not depend upon the information provided by pharmaceutical companies [25]. Continuous professional development programmes, consisting of educational meetings alone or combined with other interventions, have been shown to improve clinical practice and patient outcomes [26]. However, whether these interventions work for the practitioners of alternative systems of medicine remains to be documented. There is evidence from India [27] that education regarding zinc use can improve prescribing of ORS and zinc together. Thus, to improve adherence to treatment guidelines we recommend updating the national medical curriculum to incorporate these guidelines in all the systems of medicine. Changing behaviour by utilising private health care providers and pharmaceutical representatives, as trainers in workshops, may be critical to achieve training needs [1].

### Methodological consideration

This study included the prescriptions from providers of alternate systems of medicine and the IHPs along with the practitioners' of modern medicine to understand the treatment pattern for acute diarrhoea in children. The pharmacy assistants carried out the data collection in pharmacies and resident medical officers in the hospitals. This was done to minimise the Hawthorne effect, whereby the prescriber would have been biased in an interview or if directly observed. Pharmacies were considered the most appropriate site for data collection as most of the outpatient prescriptions reach the pharmacies for purchase of drugs. Also, pharmacy assistants have the basic information on commercial names for drugs. We built on this basic information to easily train them in completing data collection forms.

The unexpectedly high design effect affected the power of our statistical analysis, and this may have resulted in us being unable to identify other important variables, which affected the prescription patterns. We did not analyse data at the level of the individual prescriber, as the name of the prescribers was not collected for ethical reasons. We could not examine the children clinically for the degree of dehydration, systemic diseases and the status of nutrition, so we cannot comment on whether the admission or the prescription of intravenous fluids was appropriate. The study was not able to take into account the 'prescriber dispensing' of medicines at their clinics.

## Conclusions

This study showed a low adherence to standard treatment guidelines of ORS and zinc prescribing for acute diarrhoea management in children below 12 years in the city of Ujjain, India. A major public health concern is the low use of zinc and high use of antibiotics, not only by the practitioners from alternate systems of medicine and informal health-care providers, but also the specialist paediatricians who should possess a good knowledge of existing guidelines.

## Competing interests

The authors declare that they have no competing interests.

## Authors' contributions

DP, AP and CSL designed the study. DP was involved in the collection of data, analysis, interpretation of data and drafting the first manuscript. AP, GM, VD and CSL revised the paper critically for substantial intellectual content. AP and GM have been involved in the statistical analysis and interpretation of the data. VD designed the GIS map of pharmacies and hospitals. All the authors commented critically on the drafts and have given approval to the final manuscript.

## Pre-publication history

The pre-publication history for this paper can be accessed here:

http://www.biomedcentral.com/1471-2334/11/32/prepub
